# Assessing the Content and Quality of Digital Tools for Managing Gestational Weight Gain: Systematic Search and Evaluation

**DOI:** 10.2196/37552

**Published:** 2022-11-25

**Authors:** Bonnie R Brammall, Rhonda M Garad, Jacqueline A Boyle, Melanie J Hayman, Susan J de Jersey, Helena J Teede, Quoc V Hong, Alayna Carrandi, Cheryce L Harrison

**Affiliations:** 1 Monash Centre for Health Research and Implementation School of Public Health and Preventive Medicine Monash University Clayton Australia; 2 Department of Obstetrics and Gynecology Monash Health Clayton Australia; 3 Appleton Institute School of Health, Medical and Applied Sciences Central Queensland University Rockhampton Australia; 4 Department of Nutrition and Dietetics The Royal Brisbane and Women’s Hospital, Metro North Health Herston Australia; 5 Perinatal Research Centre Centre for Clinical Research, Faculty of Medicine The University of Queensland Herston Australia; 6 Diabetes and Vascular Research Monash Health Clayton Australia

**Keywords:** digital, gestational, weight, tracking, pregnancy

## Abstract

**Background:**

Digital health resources have the potential to assist women in optimizing gestational weight gain (GWG) during pregnancy to improve maternal health outcomes.

**Objective:**

In this study, we aimed to evaluate the quality and behavior change potential of publicly available digital tools (websites and apps) that facilitate GWG tracking.

**Methods:**

Digital tools were identified using key search terms across website search engines and app stores and evaluated using the Mobile App Rating Scale, the App Behavior Change Scale, as well as criteria to evaluate the rigor and safety of GWG information.

**Results:**

Overall, 1085 tools were screened for inclusion (162 websites and 923 apps), and 19 were deemed eligible. The mean Mobile App Rating Scale quality score was 3.31 (SD 0.53) out of 5, ranging from 2.26 to 4.39, and the mean App Behavior Change Scale score was 6 (SD 3.4) out of 21, ranging from 19 to 0. Of the 19 items used to evaluate rigor of GWG advice, most tools (n=11, 57.9%) contained ≤3 items.

**Conclusions:**

This review emphasizes the substantial limitations in current digital resources promoting the monitoring and optimization of GWG. Most tools were of low quality, had minimal behavior change potential, and were potentially unsafe, with minimal linkage to evidence-based information or partnership with health care.

## Introduction

### Gestational Weight Gain: An Overview

During pregnancy, gestational weight gain (GWG) is essential to ensure the development of a healthy fetus [1]. However, GWG below or above the recommendations is associated with an increased risk of negative pregnancy outcomes and neonatal conditions or complications [1-6]. Epidemiological data in over 1 million pregnancies globally reported GWG below or above the recommended thresholds in 23% and 47% of all pregnancies, respectively [7]. The associated risks of GWG below recommendations include preterm birth and the delivery of a small-for-gestational-age infant, whereas excessive GWG above recommendations was associated with cesarean section, macrosomia, and the delivery of a large-for-gestational-age infant [6-8]. Long-term, excessive GWG is associated with intergenerational adverse health risks, including obesity, cardiovascular disease, and type 2 diabetes [1-5]. Therefore, optimizing GWG during pregnancy in line with recommendations is a global health priority.

### Digital Health Engagement During Pregnancy

Digital health, including internet-based information and mobile Health (mHealth) apps, have become popular and widely used sources of health information for pregnant women, often replacing traditional paper-based and supplementing face-to-face health professional consultations [9-12]. However, the attainment of credible internet health or mHealth information is reliant on consumer health literacy and the ability to judge the quality and accuracy of information. Given the tendency of consumers to trust digital health information [13], this is problematic, as health information is not always reliable or current and can be confusing, overwhelming, and at times potentially harmful [12].

During pregnancy, freely accessible web-based resources, including trackers, calculators, or graphs, to record and self-monitor GWG have the potential to assist women in identifying whether weight gain is outside the recommended thresholds. In conjunction with the promotion of healthy lifestyle behaviors, these web-based resources have the potential to assist women in achieving healthy GWG [14-16]. However, there is currently limited information about the type of tools available, their format (ie, web-based application or mobile app) and functionality, credibility of the information provided, or their ability to guide behavior change to positively impact GWG. Evaluating digital tools that are publicly available to women to monitor GWG during pregnancy is a critical gap to address, given the risk of complications associated with excessive or inadequate GWG and the need to ensure credible and reliable self-monitoring tools for women during this time. Previous research in this area is limited to evaluations of mobile apps only and is primarily based on functionality [17] or a narrow evaluation of selected apps based on predefined pregnancy topics [18].

In this study, we aimed to evaluate the quality and behavior change potential of publicly available digital tools (websites and apps) that facilitate GWG tracking. Given the benefits of self-weighing for weight management [16] and the high use of digital health information during pregnancy [9-11], there is a need to examine and review what is currently available to ensure that pregnant women are being provided with evidence-based information and tools that align with GWG recommendations.

## Methods

The methods of this study have been informed by previous reviews exploring the quality, features, functions, behavior change capacity, and quality of digital applications and resources [19-23].

### Systematic Search

Searches were conducted in an Australian web browser using website search engines (Google, BING, and Yahoo) and mobile app stores (Apple AppStore, iOS and Google Play, Android) using a combination of search terms emulating terms likely used by end users, including *pregnancy weight, pregnancy weight tracker, pregnancy weight gain calculator, pregnancy weight graph* (website searches), and *pregnancy weight, pregnancy weight tracker,* and *gestational weight tracker* (app searches). Search terms were developed by a multidisciplinary team comprising obstetrics and gynecology (O&G), midwifery, nursing, dietetics and nutrition, and exercise physiology. Each search term combination was entered individually in the search engine. For websites, the first 2 pages of results for each search term were screened for inclusion, similar to previous studies [19,24,25]. For apps, searches were entered into the Google Play and Apple App Store databases without any specified search categories. All the retrieved app search results were screened for inclusion. One reviewer (BRB) independently reviewed all results, with a 100% cross-check of websites and 50% cross-check of apps completed by 2 additional independent reviewers (CLH and RMG).

### Inclusion Criteria and Selection Process

Websites and apps were included according to the following criteria: publicly available or ability to download (free or paid, but with free discovery capacity); written in or available in English; title or description suggested inclusion of tools or advice or resources relating to pregnancy weight gain; and weight-tracking tool enabled multiple logs or entries of weight across pregnancy (ie, not just 1 static weight log).

Apps that met the inclusion criteria were further filtered using the following app-specific inclusion criteria: updated within 18 months from the search date, May 2021; user rating of ≥4.0 stars if ≥6 months old (apps <6 months were included irrespective of user rating) as a proxy for app popularity per previous research [21]; incorporation of a graph or chart or illustration of GWG (ie, does not merely display the weight as a numerical value); and presence of surrounding content about pregnancy health and well-being. Apps that required downloading to complete this step were screened for inclusion by 2 researchers (BRB and RMG). If the apps available on Google Play and Apple AppStore had contrasting user ratings, the higher rating was carried forward and documented in the app description results.

### Resource Evaluation

#### Overview

Eligible websites and apps were randomly allocated to 2 reviewers and independently reviewed on a mobile device. All reviewers (AC, BRB, MJH, QVH, RMG, and SJdJ) have expertise in public health and form a multidisciplinary team (ie, O&G, midwifery, nursing, dietetics and nutrition, and exercise physiology). Where the same app was available on both Google Play Store and Apple App Store, app details and descriptions were reviewed to ensure consistency across the 2 platforms and downloaded for review on an Apple device. The reviews were conducted from June to July 2021. Apps were user tested for evaluation using numerous validated scales and relevant questions (Multimedia Appendices 1-4) using a mock user profile. Each app was explored until the reviewer had familiarized themselves with the functionality and features of the app, with a user experience consistent with other studies [21]. Reviewers noted whether the app stopped functioning or whether the features were not accessible. Following the review, if there was a contradiction in reviewer responses, a third independent reviewer was assigned to resolve item or items of disagreement and establish consensus (BRB, CLH, and RMG).

Collections of user demographic and pregnancy-specific data were recorded, including username; contact details (name, email, phone, or other); date of birth or age; country of origin; gestation (due date, last menstrual cycle, or date of conception); type of pregnancy (singleton, twin, triplet, etc); parity (first, second, third, etc); and preconception weight and height.

#### GWG Criteria

To evaluate the rigor and safety aspects of GWG management information, GWG-specific criteria were developed by a multidisciplinary team (Multimedia Appendix 1). The criteria encompassed 19 items, including reference to published international guidelines for GWG (ie, National Academy of Medicine, previously Institute of Medicine [26]) with personalization according to BMI; warnings, notifications, or alerts for weight gain detected outside of recommendations; direction or advice to consult a health professional if logged GWG was outside of the recommendations; and dietary and physical activity content and the development of content in consultation with relevant health professionals (O&G, midwifery, allied health, etc).

#### Mobile App Rating Scale

The Mobile App Rating Scale (MARS) is a 23-item evaluation tool comprising 6 domains (Multimedia Appendix 2): *engagement, functionality, aesthetics, information quality, subjective quality*, and *health topic specific* [27,28]. Each item is scored using a 5-point ordinal scale, with a mean score derived for each domain. The first 4 domains, including *engagement* (ie, incorporation of interesting, customizable, and interactive—eg, sends alerts, messages, reminders, and feedback and enables sharing—features targeted at the audience); *functionality* (ie, ease of use, navigation, flow logic, and gestural design); *aesthetics* (ie, graphic design, overall visual appeal, color scheme, and stylistic consistency); and *information quality* (ie, contains high-quality information from a credible source), are combined and averaged to provide an overall app quality score out of 5. A *subjective quality* score between 0 and 20 is allocated by each reviewer. This section requires the reviewer to rate whether they would recommend the app to people who may benefit from using the app, how many times over 12 months they would use the app if it was relevant to them, whether they would be willing to pay for the app, and their overall app star rating. The *health topic–specific* domain is an optional 5-item section that can be adjusted to suit the topic area researched (ie, GWG). This domain aims to assess whether the app is likely to *increase awareness of the importance of addressing GWG, increase knowledge or understanding of GWG, change attitudes toward improving GWG, increase intention or motivation to address GWG,* and *encourage further help seeking for GWG.*

The MARS also includes an App Classification section to obtain information about technical features (Multimedia Appendix 2). These items were recorded for descriptive purposes but did not form part of the functionality rating. These features include the app rating, obtained via the Google Play or Apple App Store; the number of app downloads (derived from the Google Play Store only as of August 2021; the Apple App Store does not provide app download information, so this information is precluded); whether the digital tool presented or required agreement to terms and conditions or a disclaimer; required log-in; allowed password protection; allowed sharing to social media; allowed data export; had an app community; sent reminders; required web access to function; and whether the digital tool sent push notifications. All applicable criteria were used for website evaluation, excluding ratings and downloads.

#### The App Behavior Change Scale

The App Behavior Change Scale (ABACUS) is designed to evaluate the behavior change potential of smartphone apps and websites across 4 domains (Multimedia Appendix 3) [22,23]. These include knowledge and information (ie, customized and personal features, collection of baseline information, and consequences for continuing or discontinuing behavior); goals and planning (ie, goal setting, goal reviewing, updating or changing, and willingness for behavior change); feedback and monitoring (ie, easy-to-use self-monitoring tools and data exporting or rewards or incentives); and actions (ie, reminders, prompts or cues, planning for barriers, and assistance with distractions or avoidance).

#### Quality Evaluation

Criteria to evaluate the quality of the health-related digital tools were developed (Multimedia Appendix 4) and modified from app review studies in the field authored by our group [19,20]. The criteria include statement of purpose of the app or website; contact details provided (email, phone, or fax); ownership disclosure (who owns the app or website); copyright; general disclosures; general disclaimers; advertisement disclosures; sponsorship disclosures; author or developer disclosures; author or developer credentials (credentials and affiliations); independence of sponsors or funders; references provided; and type of references provided (a list of types provided, including meta-analysis, randomized controlled trial, media, government guideline, or option piece).

### Statistical Analysis

Descriptive statistics (mean and SD) and frequencies (numbers and percentages) were calculated for all scales applied. The reported percentages were rounded to the nearest whole number. Intraclass correlation (ICC) scores were calculated to determine the agreement between the MARS rating using SPSS statistical software (version 25; IBM Corp). All analyses were conducted using SPSS for Windows, with a significance level set at *P*<.05. The following previously established categories for expressing levels of reliability for ICC results were used: high reliability, 0.90 to 0.99; good reliability, 0.80 to 0.89; fair reliability, 0.70 to 0.79; and poor reliability, 0.69 or less [29].

### Ethical Considerations

This study does not meet the criteria for human research and thus did not require oversight from the authors’ institutions.

## Results

**Figure 1:**
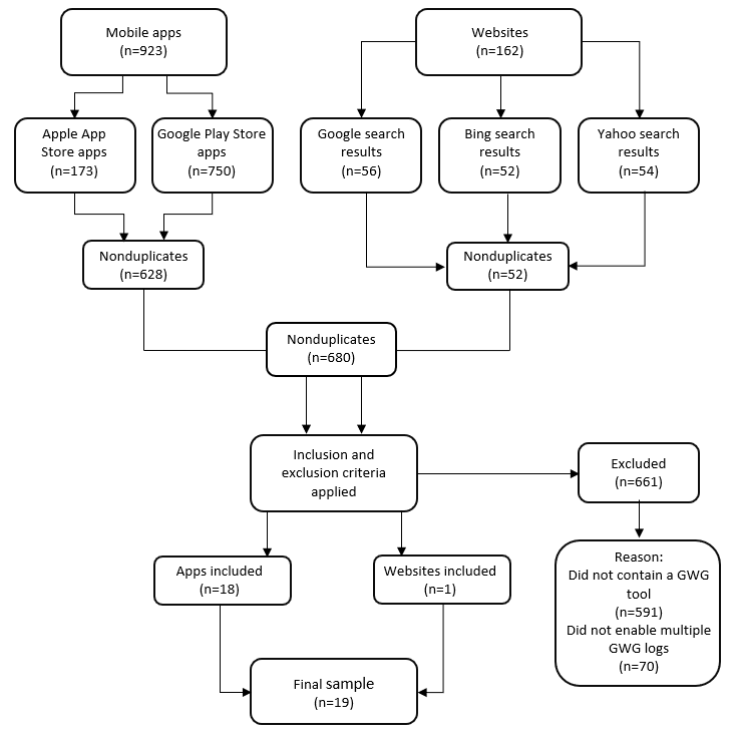
Flowchart of gestational weight gain (GWG) digital tool selection.

A total of 1085 digital tools were screened for inclusion across 162 websites and 923 apps. After excluding duplicates, 89 digital tools were retained for potential inclusion with 19 digital tools eligible for analysis (Figure 1).

### Characteristics and Overview of Digital Tools

Table 1 presents the main characteristics of the websites and apps included in this study; further descriptions of tools are available in Multimedia Appendix 5. All apps (n=18) were available on the Google Play Store and 9 were available on both the Google Play and Apple App Store. The 18 apps had a Google Play or Apple App Store user-rating score ranging from 4.10 to 4.90, with a mean score of 4.64 (SD 0.22), and had been downloaded over 25 million times from the Google Play Store alone. Most digital tools were associated with commercial enterprises (17/19, 89%), whereas few were affiliated with government services (1/19, 5%) and universities (1/19, 5%). All apps had a free discovery capacity (ability to download and use without payment), with total downloads per app ranging from >500 to >10,000,000. Overall, 50% (9/18) of apps had costs for app subscription and in-app purchases ranging from Aus $1.99 to $79.99 (US $1.49 to $59.99; Multimedia Appendix 5); however, this did not impact the discoverability of content or tools reviewed. The website (1/1, 100%) was free to access.

All digital tools were based on information or education (19/19, 100%) and monitoring or tracking (19/19, 100%), and the majority included advice, tips, and strategies (15/19, 79%). A small number of tools used assessment (3/19, 16%), feedback (3/19, 15%), and goal setting (1/19, 5%). Technical aspects included reminders (11/19, 58%), log-in requirements (11/19, 58%), app communities (5/19, 26%), password protection (4/19, 21%), and sharing options (eg, social media, app to app, or email; 3/19, 16%). Only the website required web access to function, with all apps able to be used offline. All collected information about gestation (19/19, 100%) and most, but not all, collected preconception weight (16/19, 84%) and height (14/19, 74%; Table 1).

**Table 1:** Technical aspects and characteristics of digital tools for GWG management.

			Value, n (%)		App^a^																																			Web^b^
					01		02		03		04		05		06		07		08		09	10		11		12		13		14		15		16		17		18		01
**Theoretical background or strategies**																																								
	Advice or tips or strategies or skills training	14 (74)		✓^c^		✓						✓		✓		✓		✓		✓			✓		✓						✓		✓		✓		✓		✓	
	Assessment	4 (16)						✓						✓																									✓	
	Feedback	4 (16)												✓																					✓				✓	
	Goal setting	1 (5)												✓																										
	Information or education	18 (95)		✓		✓		✓				✓		✓		✓		✓		✓		✓	✓		✓		✓		✓		✓		✓		✓		✓		✓	
	Monitoring or tracking	19 (100)		✓		✓		✓		✓		✓		✓		✓		✓		✓		✓	✓		✓		✓		✓		✓		✓		✓		✓		✓	
**Technical aspects**																																								
	Allows sharing (social media, app to app, or email)	4 (21)								✓				✓											✓														✓	
	App community	5 (26)										✓		✓		✓				✓											✓									
	Needs web access to function	1 (5)																																					✓	
	Password protected	3 (16)										✓		✓																	✓									
	Requires log-in	10 (53)				✓		✓				✓		✓									✓		✓						✓		✓		✓		✓			
	Sends reminders	11 (58)				✓		✓		✓		✓		✓				✓		✓		✓			✓				✓				✓							
**Information collected**																																								
	Contact details	9 (47)				✓				✓		✓		✓				✓					✓		✓						✓				✓					
	Country or location	4 (21)										✓		✓				✓							✓															
	Date of birth or age	6 (32)				✓						✓		✓				✓							✓				✓											
	Name	11 (58)		✓		✓				✓		✓		✓				✓							✓						✓		✓		✓		✓			
**Pregnancy-related information collected**																																								
	Gestation	19 (100)		✓		✓		✓		✓		✓		✓		✓		✓		✓		✓	✓		✓		✓		✓		✓		✓		✓		✓		✓	
	Height	14 (74)		✓				✓				✓		✓				✓		✓		✓	✓				✓				✓		✓		✓		✓		✓	
	Pregnancy number (ie, first or second etc)	4 (21)				✓						✓		✓				✓																						
	Pregnancy type (single or twins etc)	3 (16)										✓		✓																									✓	
	Preconception weight	15 (79)		✓		✓		✓				✓		✓				✓		✓		✓	✓				✓				✓		✓		✓		✓		✓	

^a^App: apps included in ths study.

^b^Web: website included in this study.

^c^✓: indicates technical aspects or characteristics present in the digital tool.

### GWG Criteria

Gestational weight tracking was a major feature of most digital tools, displayed prominently to users (15/19, 79%), in line with our inclusion criteria (Multimedia Appendix 6). In total, 58% (11/19) of digital tools provided weight recommendations based on preconception weight and height. All other criteria were present in less than half of the digital tools. Overall, 47% (9/19) of tools encouraged an unspecified, healthy diet for optimal GWG, and 37% (7/19) encouraged nonspecific, regular moderate physical activity for optimal GWG. Very few (2/19, 11%) tools alerted the user when their weight gain was outside of the recommended range, and none directed the user to consult a health professional if their weight entry was outside the recommended range. Overall, of the 19 criteria, the majority (11/19, 58%) contained ≤3 items, with 11% (2/19) having 0 items. The tool that met the most criteria for GWG was Web01 (9 of 19 criteria), followed by App17 (7 of 19 criteria) and App06 (6 of 19 criteria); the name and description of tools can be viewed in Multimedia Appendix 5. Refer to Multimedia Appendix 1 for the complete GWG criteria and definitions.

### MARS Results

The specific MARS scores for each digital tool are presented in Table 2. The overall mean MARS quality score (comprising engagement, functionality, aesthetics of tool, and the quality of general pregnancy-related information domains) ranged from 2.26 (lowest-rated app) to 4.39 (highest-rated app), with a mean score of 3.17 (SD 0.75). Subjective ratings (ie, reviewer recommendations, rating, and perceived monetary value) ranged from mean 3.25 (SD 0.00) to mean 15.50 (SD 0.71), from a potential score of 20; app-specific ratings (ie, GWG awareness, knowledge, and understanding of GWG; attitudes toward improving GWG; intention and motivation to address GWG; and help seeking for GWG) ranged from 1.00 to 4.50, with most (15/19, 79%) scores being 2.50 or less. Overall, the best-rated section was functionality (mean 3.94, SD 0.63), followed by aesthetics (mean 3.61, SD 0.69) and engagement (mean 3.19, SD 0.63), compared with app-specific (mean 2.24, SD 0.84) and information (mean 2.49, SD 0.68) domains, which scored the lowest. ICC scores ranged from 0.671 (95% CI −0.169 to 0.946) to 0.996 (95% CI 0.076-0.999). Most ICC results showed either high (10/19, 53%) or good (6/19, 32%) reliability.

**Table 2:** Mobile App Rating Scale (MARS) scoring.

App or website name	Overall MARS quality score (A-D), mean (SD)	A (engagement), mean (SD)	B (functionality), mean (SD)	C (aesthetics), mean (SD)	D (information), mean (SD)	E (subjective), mean (SD)	F (app specific), mean (SD)	ICC^a^ (95% CI)
App06	4.39 (0.54)	4.50 (0.71)	4.50 (0.71)	4.50 (0.24)	4.07 (0.51)	15.50 (0.71)	2.70 (2.40)	0.935 (0.615 to 0.991)
App12	4.07 (0.15)	3.80 (0.57)	4.75 (0.35)	5.00 (0.00)	2.72 (0.40)	13.00 (0.00)	2.30 (0.42)	0.973 (0.824 to 0.996)
Web01	4.00 (0.18)	3.20 (0.28)	5.00 (0.00)	4.00 (0.47)	3.79 (0.91)	12.50 (3.54)	4.50 (0.14)	0.836 (0.222 to 0.975)
App08	3.60 (0.04)	4.10 (0.14)	4.00 (0.00)	4.00 (0.00)	2.29 (0.00)	3.25 (0.00)	2.60 (0.57)	0.996 (0.976 to 0.999)
App02	3.56 (0.11)	3.30 (0.42)	4.13 (0.88)	4.34 (0.47)	2.50 (0.51)	8.00 (1.41)	2.00 (0.57)	0.858 (0.349 to 0.981)
App01	3.54 (0.01)	3.10 (0.14)	4.25 (0.00)	4.00 (0.00)	2.79 (0.10)	8.50 (0.71)	2.50 (0.42)	0.972 (0.817 to 0.996)
App14	3.51 (0.24)	3.50 (0.14)	3.50 (0.35)	4.34 (0.94)	2.72 (0.21)	10.00 (2.83)	2.30 (0.99)	0.856 (0.285 to 0.978)
App05	3.41 (0.06)	3.70 (0.14)	3.88 (0.18)	3.33 (0.00)	2.72 (0.21)	11.50 (0.71)	3.20 (0.28)	0.972 (0.999 to 0.817)
App03	3.39 (0.27)	3.10 (0.42)	4.25 (0.35)	4.00 (0.00)	2.22 (0.30)	7.50 (2.12)	1.70 (0.42)	0.873 (0.349 to 0.981)
App17	3.38 (0.06)	3.60 (0.00)	4.00 (0.00)	3.50 (0.24)	2.43 (0.00)	12.50 (0.71)	3.50 (0.14)	0.995 (0.962 to 0.999)
App09	3.34 (0.08)	3.20 (0.57)	3.63 (0.53)	3.33 (0.00)	3.22 (0.30)	9.50 (0.71)	2.60 (0.57)	0.957 (0.729 to 0.994)
App15	3.25 (0.10)	3.50 (0.71)	3.50 (0.00)	3.50 (0.24)	2.50 (0.10)	10.00 (1.41)	1.60 (0.28)	0.950 (0.693 to 0.993)
App07	3.14 (0.41)	3.20 (0.28)	3.88 (0.18)	3.50 (1.17)	2.00 (0.00)	7.00 (1.41)	1.60 (0.57)	0.859 (0.296 to 0.979)
App11	2.96 (0.69)	2.90 (0.71)	3.50 (0.71)	3.00 (0.95)	2.43 (0.40)	5.50 (2.12)	1.70 (0.14)	0.711 (−0.095 to 0.954)
App10	2.85 (0.62)	2.10 (0.14)	4.38 (0.88)	3.33 91.41)	1.57 (0.00)	6.00 (2.83)	1.30 (0.42)	0.713 (−0.090 to 0.954)
App13	2.84 (0.01)	2.60 (0.57)	3.88 (0.18)	3.00 (0.00)	1.86 (0.40)	7.00 (0.00)	1.90 (0.14)	0.972 (0.815 to 0.996)
App16	2.75 (0.02)	2.80 (0.00)	3.13 (0.18)	2.84 (0.23)	2.22 (0.50)	5.50 (0.71)	1.50 (0.71)	0.864 (0.315 to 0.980)
App18	2.60 (0.83)	2.50 (0.99)	3.63 (0.53)	2.50 (0.71)	1.79 (1.11)	6.50 (3.54)	2.00 (1.13)	0.671 (−0.169 to 0.946)
App04	2.26 (0.26)	2.00 (0.57)	3.13 (0.18)	2.50 (0.24)	1.43 (0.40)	4.00 (0.00)	1.00 (0.00)	0.938 (0.627 to 0.991)

^a^ICC: intraclass correlation; agreement between reviewers (A-F).

### ABACUS Results

The overall ABACUS score was 6 (SD 3.6) of 21 (Table 3). Four behavior change techniques were most prominent, which were included in >50% of the apps. These techniques or functions included the ability to customize and personalize some features (19/19, 100%), the collection of baseline information (ie, user information or personal details; 16/19, 84%), allowing the user to easily self-monitor behavior (13/19, 68%) and providing instructions on how to perform a behavior (10/19, 53%). These and other didactic or simple techniques such as instructions, data export, and sending of reminders were much more frequent than interactive functions such as goal setting (1/19, 5%), encouragement (0/19, 0%), providing material or social rewards (0/19, 0%), and ascertaining willingness to change (0/19, 0%). The top tools for behavior change potential were App06 (scoring 16/21), App08 (scoring 11/21), App17 (scoring 9/21), and Web01 (scoring 9/21).

**Table 3:** Performance on App Behavior Change Scale (ABACUS) criteria (most to least frequently used).

Behavior change technique^a^	Value, n (%)
Customize and personalize some features	19 (100)
Baseline information	16 (84)
Allow the user to easily self-monitor behavior	13 (68)
Provide instruction on how to perform the behavior	10 (53)
Reminders or prompts or cues for activity (on app)	8 (42)
Data export	7 (37)
Information provided about the consequences of continuing or discontinuing behavior	7 (37)
Give user feedback (person or automatic)	5 (26)
Allow or encourage practice or rehearsal in addition to daily activities	4 (21)
Created with expertise or information consistent with national guidelines	4 (21)
Restructure the physical or social environment	4 (21)
Encourage positive habit formation	3 (16)
Provide the opportunity to plan for barriers	3 (16)
Share behaviors with others or allow for social comparison	3 (16)
Understand the difference between current action and future goals	3 (16)
Distraction or avoidance	2 (11)
Review goals, update, and change	2 (11)
Goal setting	1 (5)
Provide general encouragement	0 (0)
Material or social reward or incentive	0 (0)
Willingness for behavior change	0 (0)

^a^App Behavior Change Scale average score: mean 6 (SD 4) out of 21.

### Quality Evaluation

Most (16/19, 84%) digital tools had a statement of purpose and all, with the exception of one (18/19, 95%), provided developer or author contact details. Ownership disclosure and copyright statements (14/19, 78%), advertisement disclosure (13/19, 68%), and author or developer disclosure (12/19, 63%) were present in most of the digital tools. No tool provided information to ascertain the independence of sponsors or funders (0/19, 0%); 5% (1/19) provided a sponsorship disclosure and 11% (2/19) outlined author or developer credentials, which included academics and O&G. Overall, 21% (4/19) of digital tools contained references (Multimedia Appendix 7). App06 met the most quality criteria (14 of 21), followed by App05 (9 of 21), and Web01 (9 of 21).

## Discussion

### Principal Findings

Women are increasingly engaging with digital resources for health guidance, including healthy lifestyles and weight gain during pregnancy. A systematic search approach identified current and publicly available websites and mobile apps that contain tools and resources to monitor GWG. Those included were reviewed based on their quality, features and functions; behavior change potential; the credibility, quality, and safety of the health-related information provided; and their ability to highlight the importance of optimizing GWG. Across 19 eligible digital tools, we found that the majority reported features including pregnancy-related education, advice, monitoring, and tracking of GWG. Despite this, the quality of information related to GWG was poor, and limited ability to guide behavior change for optimized GWG was found. Advice related to achieving healthy GWG was present in ≤50% of the apps. Overall, this advice was nonspecific in nature and unlikely to be associated with evidence-based information. We found minimal likelihood of resources to alert, provide support, or direct women into partnerships with their health care provider if GWG was outside the recommended thresholds. These results emphasize a missed opportunity in information provision and support to safely optimize health behaviors and GWG for women. There is a critical need to improve the quality and regulation of publicly accessible web-based resources informed by health care, policy, and consumer needs during pregnancy.

Pregnancy presents a unique opportunity in which women are motivated to optimize lifestyle behaviors to ensure favorable health outcomes for themselves and their baby [30]. Optimizing diet, physical activity, and ultimately GWG during pregnancy reduces adverse outcomes for mother and baby and confers protective maternal and intergenerational benefits [30-32]. Our results support a mobilization of women during this time in engagement with health resources, with over 25 million downloads observed across the 18 apps included in this review. Associated consumer user ratings for apps were very high; however, it is not clear what aspects were most appealing and why. Recent qualitative research exploring consumer preferences and experiences with mHealth apps for maternal health reported that functionality and technical ability features were perceived to be of highest value to women [33]. Consumers reported an increased likelihood to use apps that were free or low-cost, aesthetically pleasing, and with minimal technological barriers [33]. However, little emphasis was placed on the quality or credibility of information by consumers when prompted, and there was little desire to obtain and ensure evidence-based information was received [33]. This may potentially explain the high user ratings of the apps included here. On evaluation, MARS domains related to visual appeal, engagement, and functionality scored the highest overall compared with domains related to content specificity, in line with previous research evaluating pregnancy-related apps [18]. Interestingly, although not captured on the scales applied in this study, we observed that functionality was impeded in several apps by mandatory viewing of advertisements contingent on accessing free features, information, or moving between pages. However, it is unclear whether this impacted the highly scored user ratings overall.

In the absence of availability of a framework to evaluate safety features within web-based resources, we built on our previous research [19,20] and included a checklist to rigorously evaluate the presence of features related to GWG management. These included consultation with relevant health care providers in content development, linkage to clinical practice guidelines for pregnancy care and guidelines for GWG, evaluation of surrounding content to promote healthy GWG, and in-built alerts if GWG entries are outside of the recommended range. Overall, we found that only 10% disclosed development in consultation with O&G expertise, 10% used adequate referencing for GWG guidelines, 10% included an alert for GWG outside of recommendations based on preconception weight and height, and none advised health care consultation if GWG was outside of recommendations. These results emphasize a near-complete absence of components related to safety within currently available web-based resources, mandating a critical need to improve regulatory control in this field [34,35]. Previous research in over 1 million pregnancies worldwide demonstrated an increase in adverse outcomes for both mother and baby when GWG is below or above international recommendations, compared with GWG within recommended thresholds [7]. Level 1 evidence demonstrates optimized GWG and improved maternal outcomes following antenatal lifestyle intervention, and there is now a strong mandate for the implementation of effective strategies in routine care [36]. With increased engagement in and availability of resources to monitor GWG, it is essential that evidence-based information and recommendations are made available to support women, with effective translation of health information congruent with the current guidelines to minimize potential harm.

Using the validated ABACUS framework, we evaluated the capacity of the included apps to guide and support behavior change [23] toward the optimization of GWG. Our results demonstrate that beyond the ability to personalize adaptable features within apps with user information or personal data, scores for the capacity to change behavior were poor overall. Behavioral techniques common to healthy lifestyle change [37], including goal setting, problem solving, provision of consequences related to the target behavior, habit formation, and social and environmental antecedents of behavior, were rarely present. This is reflective of findings within previous non–pregnancy-related research [38] and pregnancy-related research specific to exercise and physical activity [21]. Further research is needed to fully elucidate which behavioral components embedded within web-based resources are effective in changing behavior [38]. This is particularly significant in the context of the burgeoning availability and use of health apps, yet for developers minimal evaluation of efficacy in changing health behaviors or improving health outcomes is required [38].

Altogether, our results highlight several areas of concern, culminating in a missed opportunity to support and guide women during this formative life phase of increased health care needs. First, despite increasing awareness, there is little regulatory control currently in place for digital health resources that are publicly available, which is an area warranting improvement. A recent Australian review highlighted the complexities between developer and consumer considerations and the involvement of multiple, siloed sectors, traversing medical, privacy, advertising, finance, and digital content as barriers to improving regulations to ensure consumer safety [39]. Of the policy documentation available, the review found a focus on the commercial loss or gains related to regulation over and above consumer safety, with consumers ultimately assigned as the primary evaluator in selecting safe and credible apps [39]. Given that women may base their engagement on functionality and aesthetics aspects within apps [33], there is a need to develop resources that can inform women about the quality, credibility, and safety of apps in a reliable, easy, and transparent way. This could include independent certification or endorsements not dissimilar to currently available entities, such as Health on the Net or similar [21,40]. Second, given that resources were likely to be more based on function and aesthetics, it is not unreasonable to conclude that entertainment and gamification came at the expense of credible information and support for women. Frequent exposure to advertisements highlights the potential for exploitation of women when using resources with exposure to potentially harmful information and imagery, underscoring the need for improved regulation and distinction between apps for entertainment and those for health information provision. Finally, in improving content quality within apps, improved partnership among commercial developers, policy makers, the health care sector, and with women, the consumers, at the forefront is required. Co-design of resources must occur to ensure a balance between the valued consumer attributes of apps alongside evidence-based information and effective behavior change techniques delivered in a way that women value as engaging, trustworthy, and safe. Previous research suggests that involving relevant expertise in app development does not compromise user downloads of apps, suggesting that quality can be optimized without compromising popularity [41].

### Strengths and Limitations

This study had several strengths and limitations. To ensure that we captured the available digital health resources for GWG, we used a robust search strategy across both websites and mHealth apps with minimal exclusion criteria, reflective of our search results. By reviewing current digital tools using the validated MARS and ABACUS tools, questions specific to GWG as well as evaluation of credibility of health-related information, we were able to evaluate technical features and quality as well as the behavior change potential and health information. We applied safety criteria specific to GWG management based on our previous publications [19,20] and tested all weight trackers for their ability to digitally summarize GWG, provide personalized feedback according to GWG, and alert and direct women to health care provision if GWG was outside recommendations. Owing to inconsistent search terms used for pregnancy and weight management across Google Play Store and Apple App Store, it is possible that some apps may have been inadvertently missed. Furthermore, a search for digital resources cannot be replicated due to the rapidly changing market and time-dependent popularity, which warrants the need for the development of validated search frameworks in this field.

### Conclusions

This review emphasizes the substantial limitations in publicly available consumer-facing digital resources for monitoring and optimizing GWG. Most tools reviewed were of low quality overall, had minimal ability to support behavior change, and were potentially unsafe, with minimal linkage to evidence-based information or partnership with health care. When women require increased support for health optimization, these results emphasize the minimal likelihood of currently available resources to positively influence GWG or, ultimately, health outcomes during this time. Owing to the extensive use of publicly available digital tools, these findings underscore the critical need for better linkage among health, research, and commercial sectors to design apps that are high quality across visual appeal, functionality, credibility, safety, and effectiveness in lifestyle modification and self-management of GWG.

## Appendix

Multimedia Appendix 1 Gestational weight gain criteria.

Multimedia Appendix 2 Mobile App Rating Scale.

Multimedia Appendix 3 App Behavior Change Scale.

Multimedia Appendix 4 Quality evaluation criteria.

Multimedia Appendix 5 Description of digital tools for gestational weight gain management (results table).

Multimedia Appendix 6 Performance on gestational weight gain (GWG) quality questions (inclusion of GWG-specific tools or features; results figure).

Multimedia Appendix 7 Performance on quality evaluation (results table).

## Abbreviations

ABACUS: App Behavior Change Scale
GWG: gestational weight gain
ICC: intraclass correlation
MARS: Mobile App Rating Scale
O&G: obstetrics and gynecology
