# Supplementary material for: Assessing the Content and Quality of Digital Tools for Managing Gestational Weight Gain: Systematic Search and Evaluation
Source: J Med Internet Res. 2022 Nov 25;24(11):e37552. doi: 10.2196/37552 (PMC9736757; doi:10.2196/37552)
Supplement: Multimedia Appendix 1 [file jmir_v24i11e37552_app1.docx]

###### Multimedia Appendix 1. Gestational weight gain criteria

| Question | Definition | Further information/example | Answer options. |
| --- | --- | --- | --- |
| 1. Is GWG tracking a major feature of the app/website? | Weight logging/tracking is prominent. |  | -Yes  -No |
| 2. Is supportive information about GWG a major feature of the app? | Information/feedback relating to the weight tracker is easy to access and seamlessly links to the tracker. |  | -Yes  -No |
| 3. Is the inclusion of a weight tracker in the app/website likely to highlight the importance of GWG to the user? | The inclusion of weight tracking in the app clearly demonstrates and communicates the importance of healthy GWG. |  | -Yes  -No |
| 4. Does the app/website clearly chart the user's weight against the GWG recommendations? | Clearly shows the recommended range of GWG based on the user's preconception BMI and map the user's weight entries against this. |  | -Yes  -No |
| 5. Does the app/website alert that weight gain is outside recommendations? | i.e. a pop-up with an alert/information or a red dot, which would otherwise not be red if weight logs are within a healthy range. |  | -Yes  -No |
| 6. Does the app direct the user into consultation with a health professional if weight gain is above or below the recommendations? | The app flags unhealthy weight gain and suggests that the user should seek support for a doctor or health practitioner. | May suggest booking an appointment with their GP or discussing weight gain with their OB. | -Yes  -No |
| 6b. If yes, to above in what case does the app recommend the user seek medical advice? * |  |  | -only if GWG is above recommendations  -only is GWG is below recommendations  -if GWG is either above or below recommendations  -NA |
| 7. Does the app reference guidelines for GWG? (if a guideline other than the IOM is referenced please note in free text) |  |  | -Yes, IOM guideline is referenced  -No  -Other (free text) |
| 8. Does the app/website reference total GWG recommendations? (i.e. 11.5-16kg if Normal BMI at conception) |  |  | -Yes  -No |
| 9. Does the app/website reference rates for GWG recommendations? (i.e. weight gain recommendations per week) |  |  | -Yes  -No |
| 10. Are BMI modifications for Asian ethnicity available? |  |  | -Yes  -No |
| 11. Are there GWG modifications for twins/triplets etc.? |  |  | -Yes  -No |
| 12. Does the app encourage a healthy diet for optimal GWG? |  |  | -Yes  -No |
| 13. Does the app encourage regular moderate physical activity for optimal GWG? |  |  | -Yes  -No |
| 14. Was the app developed in consultation with O&G? |  |  | -Yes  -No |
| 16. Was the app developed in consultation with midwifery? |  |  | -Yes  -No |
| 17. Was the app developed in consultation with allied health (EP, dietitian, physio etc.)? |  |  | -Yes  -No |
| 18. Was the app developed in consultation with academics? |  |  | -Yes  -No |
| 19. Was the app developed in consultation with consumers? |  |  | -Yes  -No |
| 19. Was the app developed in consultation with ‘other’? |  |  | -Yes (please specify)  -No |

## *results for 6b were not included in the manuscript as zero (n=0, 0.0%) tools met criteria 6.
