# Supplementary material for: Assessing the Content and Quality of Digital Tools for Managing Gestational Weight Gain: Systematic Search and Evaluation
Source: J Med Internet Res. 2022 Nov 25;24(11):e37552. doi: 10.2196/37552 (PMC9736757; doi:10.2196/37552)
Supplement: Multimedia Appendix 3 [file jmir_v24i11e37552_app3.docx]

**Multimedia Appendix 3. App Behavior Change Scale**

| Item | Question | Definition | Example or further information | Response: Present (yes/no) |
| --- | --- | --- | --- | --- |
| 1.1 | Does the app have the ability to customize and personalize some features? | Elements of the app can be personalized through specific tools or functions that are specific to the individual using the app. | - To select a disease type from among several available and then to follow a specific path or set of tools or systems. - To select to receive emails or texts of a specific nature. - To choose “yes” or “no” to a specific capability of the app would be considered personalization. - To create a personalized exercise plan. |  |
| 1.2 | Was the app created with expertise and/or Does the app provide information that is consistent with national guidelines?  (Are the most recent IOM guidelines referenced?) | This would be found in the about section or generally in the app. | - Does the app suggest 30 min of exercise each day? - Does it recommend 5 veg and 3 fruit? - Does it seek to build resilience and promote help seeking? - Is there any evidence that the app was created by an expert? (doctor/professional body/university)‎ |  |
| 1.3 | Does the app ask for baseline information? | This includes BMI^a^, weight, smoking rate, exercise, or drinking behaviours | - This might be at the set-up phase or in a profile setting. |  |
| 1.4 | Does the app provide instruction on how to perform the behaviour? | The app is clear in telling the person how to perform a behaviour or preparatory behaviours, either verbally, through video, or in written form. NB: the behaviour that is seeking to be changed (weight gain above or below recommendation), not information on how to use the app | - This could include recipes, meals planes or structured advice such as showing person how to use gym equipment, sharing sample plans for action.   ‎ |  |
| 1.5 | Does the app provide information about the consequences of continuing and/or discontinuing behaviour? | The app gives the user information about the consequences of behaviour in general, this includes information about the relationship between the behaviour and its possible or likely consequences in the general case. This information can be general or personalized. | - Consequences may include health risks to mother and baby and/or informing on decreased risks of complications (LGA, SGA, CS, GDM) if GWG is within recommendations ‎ |  |
| 2.1 | Does the app ask for willingness for behaviour change? | Is there a feature during setup where you describe how ready you are for behaviour change? | - This may be in the form of a scale of readiness or in a question that asks the user to describe how ready you are. |  |
| 2.2 | Does the app allow for the setting of goals? | The person is encouraged to make a behavioural resolution. The person is encouraged to set a general goal that can be achieved by behavioural means. This includes sub goals or preparatory behaviours and/or specific contexts in which the behaviour will be performed. The behaviour in this technique will be directly related to or be a necessary condition for the target behaviour. | - This is the explicit noting of a goal or choosing a goal from one provided within the app. |  |
| 2.3 | Does the app have the ability to review goals, update, and change when necessary? | Involves a review or analysis of the extent to which previously set behavioural goals (regardless of short or long) were achieved. | - This is where a goal can be changed. This allows people to act on previously set goals and then revise or adjust where needed. |  |
| 3.1 | Does the app give the user the ability to quickly and easily understand the difference between current action and future goals? | Allows user to see how they are tracking against a goal and to see the difference between what they want to do and what they are currently doing. This will give some feedback on where they are at and what they need to change to get to where they want to be. | - This could be in the form of a graph or some other visual describing how close the user is to meeting their goals.   ‎ |  |
| 3.2 | Does the app have the ability to allow the user to easily self-monitor behaviour? | The app allows for a regular monitoring of the activity. | - Allows for tracking of weight gain. - Connects with watch that records daily steps that can be reviewed. - Allows for easy logging of exercise or meditation. - Allows tracking of food intake. |  |
| 3.3 | Does the app have the ability to share behaviours with others (including social media or forums) and/or allow for social comparison? | The app allows the person to share his or her behaviours on social media or in forums. This could also include a *buddy* system or a leader board. | - Share with Facebook or other socials‎ - Tell the user that they are doing x and at this time, other people like them are doing y (comparative behaviour) |  |
| 3.4 | Does the app have the ability to give the user feedback—either from a person or automatically? | The app is able to provide the person with feedback, comments, or data about their own recorded behaviour. This might be automatic or could be personal. | - Does the app have a *coach* function?   ‎ |  |
| 3.5 | Does the app have the ability to export data from app? | The app allows for the export of information and progress to an external user. | - Export to a computer or to another user such as a doctor or fitness expert. - Sharing to Facebook does not count. |  |
| 3.6 | Does the app provide a material or social reward or incentive? | App provides rewards for attempts at achieving a behavioural goal. This might include efforts made toward achieving the behaviour or progress made in preparatory steps toward the behaviour or in achieving a goal. | - Financial, either in returning money that was not spent on, for example, cigarettes or in paying someone to engage in a specific activity. - Social or public, for example, congratulating the person for each day that he or she meets his or her exercise target. |  |
| 3.7 | Does the app provide general encouragement? | The app provides general encouragement and positive reinforcement on actions leading to the goal. | - This could include achievement badges or telling the user that they are a certain percentage closer to their goal. |  |
| 4.1 | Does the app have reminders and/or prompts or cues for activity? | The app prompts the user to engage in the activity. The app has the ability to give notifications or reminders to cue the behaviour. | - This could be like the apple watch reminding you to exercise or to log your weight. |  |
| 4.2 | Does the app encourage positive habit formation? | The app prompts explicit rehearsal and repetition of the behaviour–not just tracking or logging. | - An example of this are the couch to 5 km apps that provide a training schedule. |  |
| 4.3 | Does the app allow or encourage for practice or rehearsal, in addition to daily activities? | App does not have a lock on activities or a number that you cannot exceed daily. | - This would include allowing the user to undertake extra activities in a single day. |  |
| 4.4 | Does the app provide opportunity to plan for barriers? | The app encourages the person to think about potential barriers and identify ways of overcoming them. | - Might give strategies for cravings or night time food indulgences.   ‎ |  |
| 4.5 | Does the app assist with or suggest restructuring the physical or social environment? | The app prompts the person to alter the environment in ways so that it is more supportive of the target behaviour. | - Might suggest locking up or throw away or their high-calorie snacks or take their running shoes to work.‎ |  |
| 4.6 | Does the app assist with distraction or avoidance? | The app gives suggestions and advice on how the person can avoid situations or distract themselves when trying to reach their goal. | - For example, may suggest that the user not eat chocolate if it is associated with overeating or cravings. ‎ |  |
