# Supplementary material for: Assessing the Content and Quality of Digital Tools for Managing Gestational Weight Gain: Systematic Search and Evaluation
Source: J Med Internet Res. 2022 Nov 25;24(11):e37552. doi: 10.2196/37552 (PMC9736757; doi:10.2196/37552)
Supplement: Multimedia Appendix 4 [file jmir_v24i11e37552_app4.docx]

**Multimedia Appendix 4. Quality evaluation criteria**

Does the app/website or app store description/developer website state and/or clearly display the following:

|  | Present (yes/no) |
| --- | --- |
| Statement of purpose of the app/website |  |
| Contact details provided (email or phone/fax) |  |
| Ownership disclosure (who owns the app/website) |  |
| Copyright |  |
| Advertisement disclosures |  |
| Sponsorship disclosures |  |
| Author/developer disclosures |  |
| Author/developer credentials (credentials and affiliations) |  |
| Independence of sponsors/funders |  |
| References provided |  |
| Type of references provided   - Meta-analysis - Systematic review - Narrative review - Scoping review - RCT - Cohort or cross-sectional study - Opinion piece - Grey literature (i.e. media) - Government Guidelines - Position statement - Medical Textbook |  |
