# Supplementary material for: Assessing the Content and Quality of Digital Tools for Managing Gestational Weight Gain: Systematic Search and Evaluation
Source: J Med Internet Res. 2022 Nov 25;24(11):e37552. doi: 10.2196/37552 (PMC9736757; doi:10.2196/37552)
Supplement: Multimedia Appendix 5 [file jmir_v24i11e37552_app5.docx]

**Multimedia Appendix 5. Description of Digital Tools for GWG Management (results table)**

| **Manuscript Reference** | **App name/website** | **Platform** | **Developer** | **User rating** | **Installations** | **Cost (AUD)** | **Affiliation** |
| --- | --- | --- | --- | --- | --- | --- | --- |
| App01 | Pregnancy Tracker, Week by Week, Day by Day | Android | Timskiy | 4.9 | 500,000+ | $0.00 - $16.99/ in-app item | Commercial |
| App02 | Pregnancy + \| tracker app, week by week in 3D | iOS/Android | Health & Parenting Ltd | 4.8 | 10,000,000+ | $0.00 - $5.99 | Commercial |
| App03 | Pregnancy app (iOS), Pregnancy Week by Week (Android) | iOS/Android | Amila | 4.8 | 5,000,000+ | $0.00 | Commercial |
| App04 | 280days: Pregnancy Diary | iOS/Android | Amane Factory Inc. | 4.8 | 1,000,000+ | $0.00 - $2.99 | Commercial |
| App05 | Nurture: Pregnancy + Baby App (iOS), GLOW. Pregnancy & Baby Tracker + Baby Registry App (Android) | iOS/Android | Glow Inc | 4.8 | 1,000,000+ | $0.00 - $79.99/ subscription | Commercial |
| App06 | Ovia Pregnancy Tracker: Baby Due Date Countdown | iOS/Android | Ovia Health | 4.8 | 1,000,000+ | $0.00 | Commercial |
| App07 | MomDiary: Week by week Pregnancy Tracker | Android | High-tech solution | 4.8 | 100,000+ | $0.00 | Commercial |
| App08 | AMMA Pregnancy Tracker & Baby Due Date Calculator | Android | Period Tracker & Pregnancy and Baby Calendar | 4.7 | 5,000,000+ | $0.00 - $11.99/ subscription | Commercial |
| App09 | I'm Pregnant - Pregnancy Week by Week | Android | BabyJoyApp | 4.7 | 1,000,000+ | $0.00 - $1.99 | Commercial |
| App10 | My Pregnancy (iOS), My Pregnancy - Pregnancy Tracker App 🤰 (Android) | iOS | Neiman / Aleksei Nieman | 4.7 | 500,000+ | $0.00 | Commercial |
| App11 | Embarazo Semana a Semana | iOS/Android | Marilia SAS | 4.7 | 500,000+ | $0.00 | Commercial |
| App12 | Belly - Your pregnancy app | iOS/Android | Life of Svea AB | 4.7 | 10,000+ | $0.00 | University |
| App13 | Pregnancy Care - Pregnancy Tracker & Tips | Android | KudoMetrics Technologies Private Limited | 4.7 | 500+ | $0.00 | Commercial |
| App14 | My Pregnancy Tracker (iOS), My Pregnancy Tracker Week by Week + Due Date (Android) | iOS/Android | My Pregnancy and Baby Tracker | 4.6 | 100,000+ | $0.00 - $23.00 | Commercial |
| App15 | Pregnancy Companion - Week by Week Tracking | Android | Healthcare Apps | 4.4 | 50,000+ | $0.00 - $4.09 | Commercial |
| App16 | Pregnancy Week by Week | Android | Promotube AAC | 4.4 | 10,000+ | $0.00 | Commercial |
| App17 | PregiCare - Pregnancy Toolkits, Weekly, Daily info | Android | Flipflapp | 4.2 | 10,000+ | $0.00 - $7.99/ in-app item | Commercial |
| App18 | Pregnancy Calendar | Android | Ruthie apps | 4.1 | 10,000+ | $0.00 | Commercial |
| Web01 | Get Healthy NSW | Website | Get Healthy NSW | Not applicable | Not applicable | Not applicable | Government |
