# Supplementary figures and images for: Assessing the Content and Quality of Digital Tools for Managing Gestational Weight Gain: Systematic Search and Evaluation
Source: J Med Internet Res. 2022 Nov 25;24(11):e37552. doi: 10.2196/37552 (PMC9736757; doi:10.2196/37552)

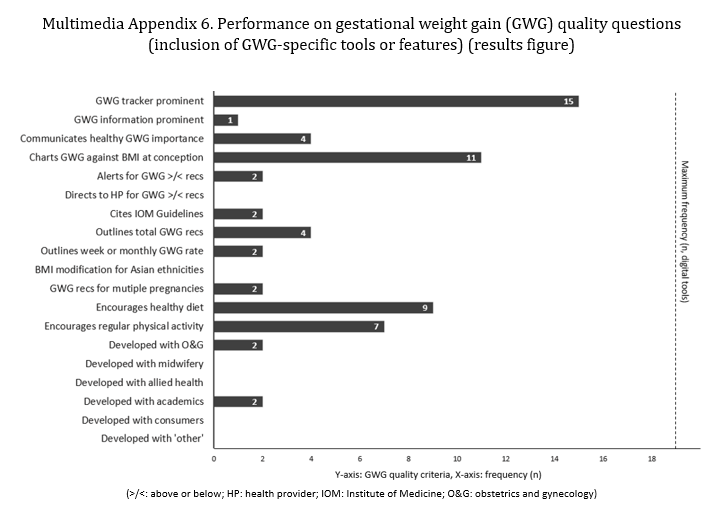

Supplement: Multimedia Appendix 6 [file jmir_v24i11e37552_app6.png]
