# Supplementary material for: Assessing the Content and Quality of Digital Tools for Managing Gestational Weight Gain: Systematic Search and Evaluation
Source: J Med Internet Res. 2022 Nov 25;24(11):e37552. doi: 10.2196/37552 (PMC9736757; doi:10.2196/37552)
Supplement: Multimedia Appendix 7 [file jmir_v24i11e37552_app7.docx]

**Multimedia Appendix 7.** **Performance on quality evaluation (results table)**

| **Criteria** | **Frequency, n (%)** |
| --- | --- |
| Statement of purpose | 16 (84.2) |
| Contact details provided (email or phone/fax) | 18 (94.7) |
| Ownership disclosure (owner of the app/website disclosed) | 14 (73.7) |
| Copyright statement | 14 (73.7) |
| Advertisement disclosure | 13 (68.4) |
| Sponsorship disclosure | 1 (5.3) |
| Author/developer disclosure | 12 (63.2) |
| Author/developer credentials | 2 (10.5) |
| Independence of sponsors/funders | 0 (0.0) |
| References provided | 4 (21.2) |
| Meta-analysis | 1 (5.3) |
| Systematic review | 1 (5.3) |
| Narrative review | 0 (0.0) |
| Scoping review | 0 (0.0) |
| Randomised control trial (RCT) | 1 (5.3) |
| Cohort or cross-sectional study | 1 (5.3) |
| Opinion piece | 0 (0.0) |
| Media | 1 (5.3) |
| Government guidelines | 2 (10.5) |
| Position statement | 0 (0.0) |
| Medical textbook | 2 (10.5) |
| **Quality evaluation, mean (SD) / total** | **5.4 (2.9) / 21** |
